# Coronary artery dilation associated with anti-synthetase syndrome in an adolescent

**DOI:** 10.1186/s12969-019-0304-y

**Published:** 2019-01-10

**Authors:** Karim Asi, Anand Gourishankar, Ankur Kamdar

**Affiliations:** Department of Pediatrics, University of Texas McGovern Medical School, 6431 Fannin St. MSB 3.228, Houston, TX USA

**Keywords:** Antisynthetase syndrome, Coronary, PL-12, Idiopathic inflammatory myopathy, Juvenile dermatomyositis

## Abstract

**Background:**

Idiopathic inflammatory myopathies (IIM) are a group of systemic autoimmune disorders primarily affecting skeletal muscle. Pediatric coronary artery dilation is frequently discussed in Kawasaki disease. However, it has yet to be reported in the IIMs or antisynthetase syndrome. We report a unique case of a patient with IIM, antisynthetase syndrome and coronary artery dilation.

**Case presentation:**

We report an adolescent presenting with joint symptoms, fever, and eye swelling with a clinical diagnosis of Juvenile Dermatomyositis. He subsequently developed diastolic hypotension with evidence of coronary artery dilation. He received steroids and immunoglobulin and followed by immunosuppressants with mild improvement in his symptoms. The adolescent later developed dyspnea and cough with CT lungs evident for cystic changes; lung biopsy showed interstitial fibrosis and inflammation, and muscle biopsy was abnormal as well. The anti-pl-12 antibody was positive. Following several weeks of treatment, an echocardiogram showed improvement in coronary artery dilation. His joint symptoms, muscle strength and respiratory symptoms have also improved.

**Conclusions:**

Coronary artery dilation is not well understood in IIMs or antisynthetase syndrome. Pathobiology of coronary artery involvement, its treatment and prognosis, and association with IIM and antisynthetase syndrome needs further exploration.

## Background

Idiopathic inflammatory myopathies (IIM) are a group of systemic autoimmune disorders primarily affecting skeletal muscle. Juvenile Dermatomyositis (JDM) is the most common childhood IIM, making up approximately 85% of cases [1]. It has a reported annual incidence of 2.5–4 cases per one million children, with a peak incidence of 5–10 years old. Females are affected at least twice as often as males [2, 3]. Multiple other organ systems may be involved; cardiovascular manifestations are less common compared to other organ systems. The current literature describes accelerated atherosclerosis and coronary artery disease, conduction abnormalities, and ventricular dysfunction as the most common cardiac complications of IIM [4–6]. To our knowledge, there is no report of IIM associated with coronary artery dilation. We present a unique case of an adolescent with IIM, anti-synthetase syndrome, and coronary artery dilation.

## Case presentation

We report a 15-year-old African-American male who presented with a six-week history of polyarthralgias, fevers, and bilateral eye and foot swelling. Initial laboratory studies revealed an elevated ALT of 337 units/L and AST 380 units/L. Infectious workup was negative. Over the next 3 weeks, he developed worsening polyarthralgias and progressive muscle weakness.

Review of systems revealed substernal chest pain while lying down, intermittent dysphagia and Raynaud’s phenomenon in his hands and feet. Physical examination revealed 4/5 proximal muscle weakness in upper and lower extremities, heliotrope rash, and telangiectasias upon nail fold capillaroscopy but no Gottron’s papules. Laboratory values included: CK 11426 units/L (19–191 units/L), aldolase> 50.0 units/L (3.4–8.6 U/L), CRP 64.5 mg/L (< 8 mg/L), ESR 77 mm/h (0–15 mm/h), positive ANA (1:640 titer, nuclear membrane pattern). MRI hip and femur revealed bilateral multifocal patchy muscular edema, most markedly within the distal gluteus medius proximally and the distal semimembranosus muscles. The patient was subsequently diagnosed with JDM based on fulfillment of Bohan and Peter criteria. He was admitted for further workup and treatment.

While admitted, prior to treatment, he developed tachycardia (96–121 bpm), with diastolic blood pressures in the 30–40s despite normal systolic blood pressures at 99–111 mmHg. The cardiovascular examination showed regular rhythm without a murmur, rub, or gallop. Echocardiogram revealed diffuse dilation of the left main coronary artery (LMCA) (5.91 mm, Z-score 4.2) as well as the left anterior descending (LAD) artery (4.42 mm, Z-score 3.1), with normal intracardiac anatomy and normal ventricular size, wall thickness, and systolic function. Electrocardiogram showed sinus tachycardia. Chest x-ray was negative, without any evidence of interstitial lung disease. He was treated with IV methylprednisolone (IVMP) 1 g daily for 4 days and intravenous immunoglobulin (IVIG) at 1 g/kg for 1 dose. Afterward, his diastolic BP improved and tachycardia resolved. CK improved to 3300 units/L. He went home on 40 mg daily prednisone, 100 mg cyclosporine twice daily, 25 mg methotrexate weekly, 1 mg folic acid daily, and aspirin 81 mg daily.

Four weeks after discharge, he developed progressive right-sided shoulder pain, shortness of breath, and dyspnea, with 10 pound weight loss. CT chest showed bilateral diffuse pulmonary infiltrate, with lower lobe predominance, with prominent parenchymal interlobular septa, ground-glass opacities, subpleural cystic changes, consistent with non-specific interstitial pneumonia pattern. Due to progressive symptoms refractory to outpatient management, he was admitted again for further workup and treatment. Subsequent lung biopsy showed severe, extensive interstitial fibrosis with a mixed inflammatory infiltrate and focal subpleural cystic change. Muscle biopsy revealed mild variation of muscle fiber size, mild atrophy of fibers, predominately type 2, and a slight increase of internal nuclei. Immunohistochemistry demonstrated positive MHC1 stain with a membranous pattern. An inflammatory infiltrate, perifasicular atrophy, calcifications, necrosis, fibrosis or tubulo-reticular inclusions were not seen. Myositis-specific antibody panel was positive for an anti-PL-12 antibody. Given the severity of symptoms, monthly cyclophosphamide IV was added to his treatment regimen, and methylprednisolone IV ‘pulses’ at 1 g IV daily for 3 days were started. Follow up echocardiogram revealed borderline dilation of the left main coronary artery (4.6 mm, Z-score of 2) and a left anterior descending coronary artery of normal size (2.9 mm). There was no evidence of coronary artery aneurysm. Three weeks after discharge, he reported symptomatic improvement with improved joint mobility, muscle strength, and no further chest pain.

## Discussion and conclusions

The anti-PL-12 antibody is one of the eight myositis-specific autoantibodies directed against aminoacyl-tRNA synthetases, associated with the anti-synthetase syndrome. This syndrome consists primarily of interstitial lung disease in addition to myositis, arthritis, Raynaud’s phenomenon, skin rashes, “mechanic’s hands,” and constitutional symptoms such as fever [7]. There is limited information on cardiac involvement in the anti-synthetase syndrome [8]. A 2011 systematic review evaluating patients with IIM with cardiac involvement had noted that it is an important cause of morbidity and mortality. The authors noted that 68 patients had pathology with 5 patients had small vessel disease and 2 patients had coronary sclerosis. Of all patients with echocardiogram findings, none were reported having coronary dilation [9]. Further, an analysis of the National Inpatient Sample by Silverberg et al. detailed the cardiovascular comorbidities associated with JDM — coronary artery disease was classified as one of the disorders that had no or too infrequent occurrences to allow for analysis [10].

It would be critical to consider other possibilities in the differential diagnosis. There is an extended rheumatologic differential diagnosis for coronary artery enlargement in pediatrics. Incomplete Kawasaki Disease (KD) was considered as he had fever> 5 days, and feet swelling without oral/mucosal changes, polymorphous rash, conjunctivitis, or cervical adenopathy. However, his age, and clinical phenotype of muscle disease, interstitial lung disease, Raynaud’s phenomenon and (+) PL-12 antibody would be inconsistent with KD. The quick resolution of his coronary dilation, given the length of time of his disease process also makes KD less likely.

Conversely, non-KD febrile illnesses associated with coronary artery dilation is noted in pediatric patients and has been noted in a variety of inflammatory and infectious causes such as systemic Juvenile Idiopathic Arthritis (JIA), Henoch Schonlein Purpura, Epstein Barr Virus, and rickettsial disease [11–14]. In a pilot prospective study evaluating pediatric patients with daily fevers > 38 °C, the authors found that in non-KD febrile patients had Z scores ranging from 2 to 2.5. Among the 43 febrile non- KD patients, 37% of the diagnosis were bacterial, 47% viral, and 16% unknown [15]. In our patient, z-scores were higher at 3.1 and 4.2 for the LAD and LMCA respectively, suggestive that his coronary artery dilation was unlikely related to infectious cause. For our patient, systemic JIA was less likely given the lack of organomegaly, evanescent rash, leukocytosis or pericarditis.

Coronary dilation, including giant aneurysms, is a well-documented, but uncommon finding in Takayasu’s Arteritis [16–18]. Our patient did not have any evidence for hypertension, claudication- type symptoms, or reduced pulses. In addition, there were no other findings on echocardiogram to suggest extended aortitis.

It is notable that his coronary dilation does not fully explain his symptoms that prompted the cardiac work up at the initial admission. Yet, it may be important to consider characterizing the cardiac phenotype of patients who present with JDM. Recently, a retrospective review of a Canadian cohort noted that in a large cohort of 52 patients with JDM, cardiac findings are common at the presentation of JDM. None of the patients were reported to have coronary dilation. The authors concluded that cardiac abnormalities were not necessarily from the disease process. However, they recommended cardiac evaluation on all patients presenting with JDM given the high number of cardiac findings and known long term cardiac dysfunction long term [5, 19].

In sum, we report a unique case of an adolescent with IIM, antisynthetase syndrome and coronary artery dilation. Patients with coronary artery dilation are at increased risk for thrombosis and, less commonly, rupture. It would be important to continue with research and investigation to better characterize the cardiac complications for patients with IIM.

## Abbreviations

IIM: Idiopathic Inflammatory Myopathy
IVIG: Intravenous Immunoglobulin
IVMP: Intravenous methylprednisolone
JDM: Juvenile dermatomyositis
JIA: Juvenile Idiopathic Arthritis
KD: Kawasaki Disease
LAD: Left Anterior Descending
LMCA: Left Main Coronary Artery
